# Global warming hiatus contributed weakening of the Mascarene High in the Southern Indian Ocean

**DOI:** 10.1038/s41598-020-59964-7

**Published:** 2020-02-24

**Authors:** Vidya P.J., M. Ravichandran, M. P. Subeesh, Sourav Chatterjee, Nuncio M.

**Affiliations:** ESSO- National Centre for Polar and Ocean Research (NCPOR), Headland Sada, Goa, 403 804 India

**Keywords:** Climate sciences, Ocean sciences

## Abstract

The Mascarene High (MH) is a semi-permanent subtropical high-pressure zone in the South Indian Ocean. Apart from its large influence on African and Australian weather patterns, it also helps in driving the inter-hemispheric circulation between the Indian Ocean in the south and subcontinental landmass in the north. Using observations and reanalysis products, this study for the first time investigates recent warming trend observed in the MH region during the Global Warming Hiatus (GWH) period (1998–2016). Significant positive trends are observed in sea surface temperature (SST), sea surface height (SSH) and oceanic heat content (OHC) during this period in the MH region. Mixed layer heat budget analysis reveals the dominant role of heat advection in the observed warming trend. During the GWH period, stronger zonal currents advect the warm waters from the Western Pacific (WP) towards the MH region via the Indonesian Throughflow (ITF). This warming in the MH reduces the sea level pressure therein and establishes a weak pressure gradient between the MH and the northern hemisphere landmass. This in-turn weakens the cross-equatorial winds in the western Indian Ocean.

## Introduction

**Figure 1:**
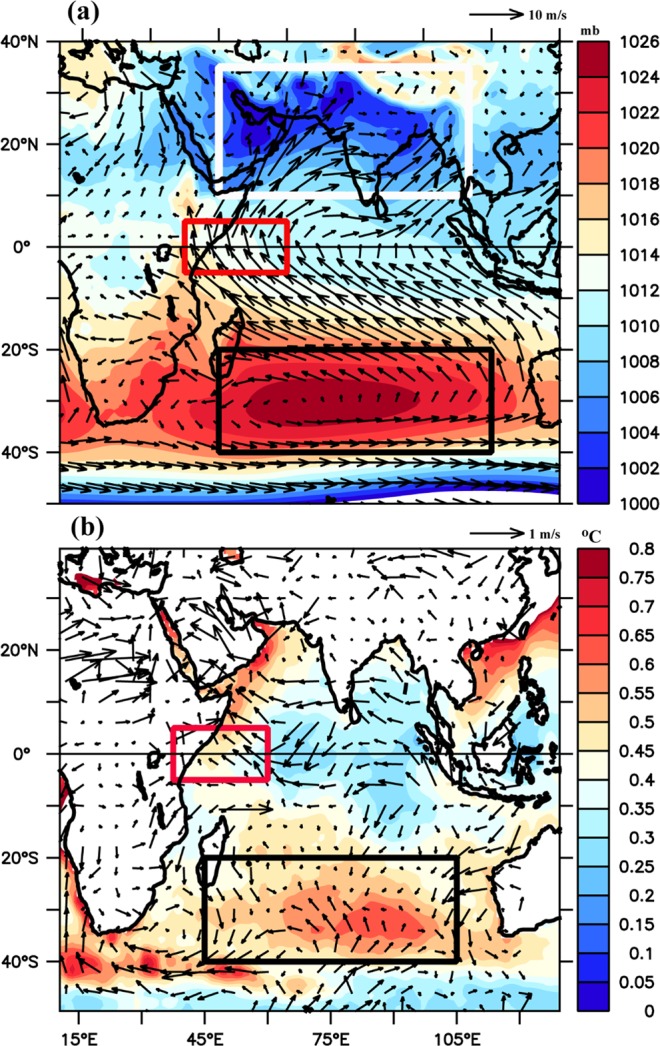
(**a**) Spatial pattern of Mean Sea Level Pressure overlaid with climatological winds (10 m) during the boreal summer (June-September), (**b**) Standard deviation of annual mean sea surface temperature overlaid with wind anomaly (GWH-preGWH). In Figure (a and b), the black rectangle in the southern hemisphere indicate the MH region [45°E–100°E and 20°S–40°S], the red rectangle indicates the cross-equatorial winds [37.5°E–60°E and 5°S–5°N]. In (**a**), the white rectangle indicates the Indian landmass [45°E–100°E and 10°N–35°N], where the SLP difference was plotted in Fig. 5b. SLP and Winds were taken from ERA-interim, and SST was taken from OISST.

The Mascarene High (MH), also called the Indian Ocean subtropical high, is a high-pressure area located between 20°S–40°S and 45°E–100°E (See Fig. 1a) near the Mascarene Islands in the Southern Indian Ocean (SIO). It undergoes strong interannual variability that largely contributes to the SST in the SIO^1^. It is also important for weather and rainfall patterns over the Australian and African landmass^2,3^. The high-pressure system intensifies during the boreal summer, and simultaneously, a low-pressure area develops in the northern hemisphere due to intense solar heating of the subcontinental landmass. This establishes a strong pressure gradient between northern and southern hemisphere and creates a cross-equatorial wind between the two regions^4^ and a strong oceanic monsoon current over the Arabian Sea. The anticyclonic circulation in the MH and its associated cross-equatorial winds in the western Indian Ocean, transport moisture from the SIO to South Asia, establishing a relationship between the MH and the Indian monsoon trough (Fig. 1a). This, in turn, affects the onset of the monsoon over the Indian subcontinent^5^ and rainfall over the east Asia^6^. The cross-equatorial winds also induces strong upwelling along the coast of Somalia and Oman^7^ which makes the region highly productive by supplying the nutrients to the upper surface layers^8^. Hence, it is imperative to understand the physical mechanisms responsible for the changes in the MH intensity and its impact on the cross-equatorial winds. Remote influences from the El Niňo-Southern Oscillation (ENSO) and the Antarctic Oscillation leads to interannual variability of SST in the MH region during the austral summer and fall^9^. These anomalous events disturb the subtropical high and change the trade winds, which modulate the surface heat flux into the ocean and generate SST anomalies. Variations in the MH intensity, due to both tropical and high latitude teleconnections, can lead to dipole response of SST in the SIO, popularly known as Indian Ocean Subtropical Dipole, which has significant impacts on Indian, African and Australian rainfall patterns^10^.

The MH intensity can also be influenced by the changes in the western Pacific (WP) owing to the Indonesian Throughflow (ITF). Since 1998, the WP has been warming which is one of the signatures of the Global Warming Hiatus, or break in 20th-century warming^11,12^. This period is characterized by a La Niña–like cooling in the tropical eastern Pacific which is accompanied by warming in the Indian and the tropical Atlantic Oceans^11,12^. Historically, there have been many periods of GWH over the years. The GWH can be triggered by the Pacific Decadal Oscillation (PDO), or by external natural forcings, such as volcanic eruption and aerosols or both^13–15^. During the GWH, the surface layer in the WP gets warmer, and this warm water is transported to the SIO through Indonesian Throughflow (ITF)^16–19^. This warming in the WP also results in the strengthening of the ITF^18,20^, which leads to increased transfer of heat from the Pacific to SIO during the GWH period^21^. Considering these changes during the GWH, and owing to teleconnection between the WP and SIO, the present study attempts to address the changes in the MH during the GWH and its possible reasons.

## Results and Discussion

**Figure 2:**
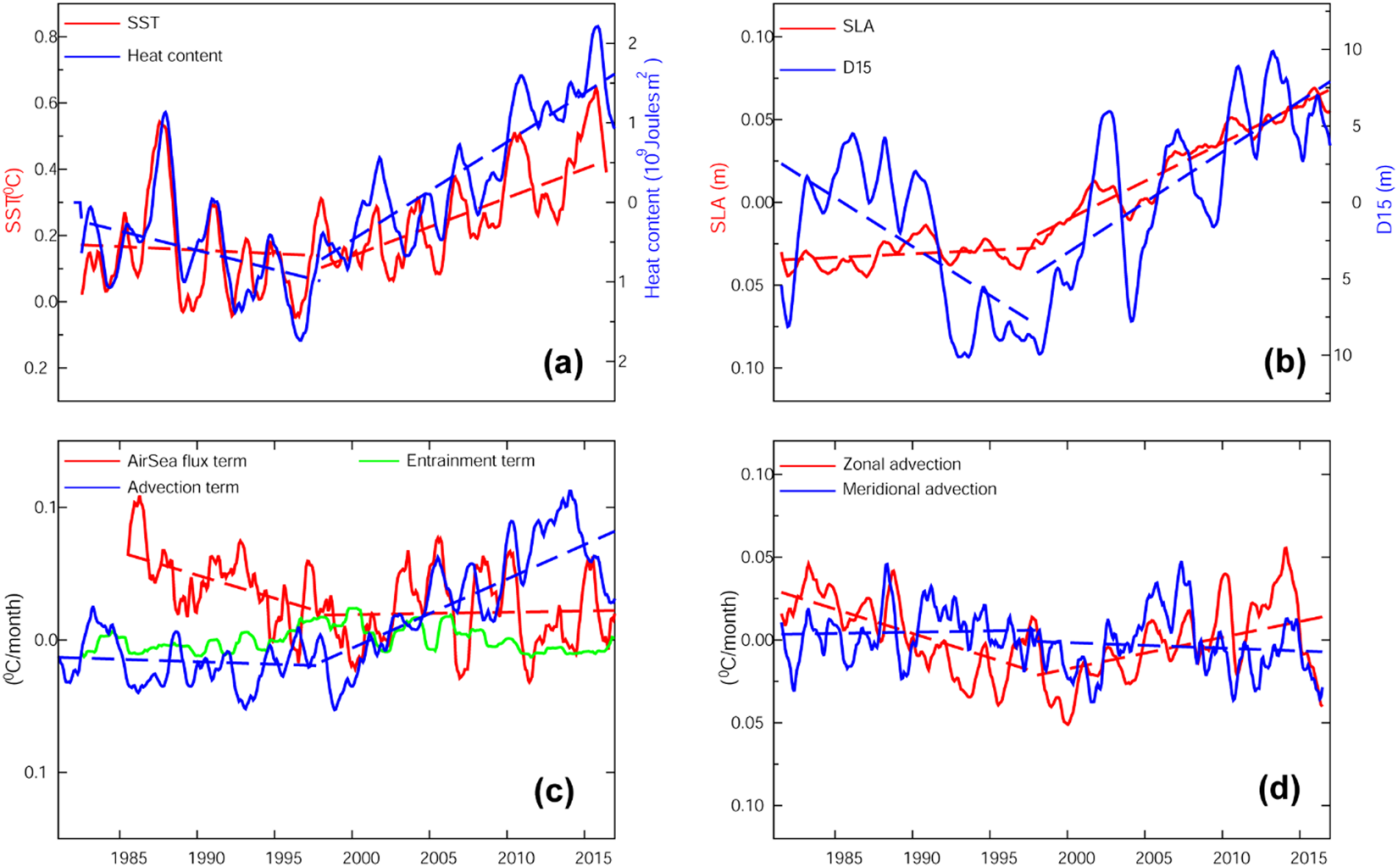
Monthly mean anomalies of (**a**) SST (red) and heat content (blue), (**b**) sea level (red), (**c**) mixed layer budget terms (Air-sea flux term - red, Advection term- blue, Entrainment term – green), (**d**) zonal (red) and meridional (blue) component of the advection term in the Mascarene High region [20°S–40°S & 45°E–100°E]. The dashed lines represent the trend line. All trends are significant above a 95% confidence level.

The standard deviation of annual mean SST shows higher value in the MH region, indicating a strong interannual variability (Fig. 1b). The study focuses on changes in the MH region since 1998, i.e., during the GWH period. For this purpose, the period before 1998 (1982–1997) is referred to as preGWH and thereafter (1998–2016) it is referred as the GWH. SST anomaly in the MH region shows a negative trend during the preGWH and positive trend during the GWH (Fig. 2a; red line). Similar to SST, upper ocean heat content (0–200 m) and sea level anomaly show a positive trend during the GWH period (Fig. 2b) confirming the warming of the upper water column during the period. In order to understand the physical processes that control the SST variability in the MH, individual components of mixed layer heat budget using Eq. (1) are computed. The anomalies (after removing the seasonal cycle) of each term are shown in Fig. 2c. Since the contribution from the diffusion to the SST variability is very low or close to zero; it is not included in Fig. 2c. Figure 2d represents the zonal (red line) and meridional advection (blue line) terms.

During the preGWH, the anomalies of SST (Fig. 2a; red line) and air-sea heat flux (Fig. 2c; red line) show a negative trend, and the advection term remains almost constant. This indicates that the decreasing trend in SST over the MH region during the preGWH (1982–1997) was controlled by the air-sea heat fluxes. On the other hand, during the GWH period, air-sea flux shows no significant trend while the advection term shows a positive trend, which confirms that horizontal advection by the ocean currents, contributed to the increase in SST in the MH region (Fig. 2a; red line).

**Figure 3:**
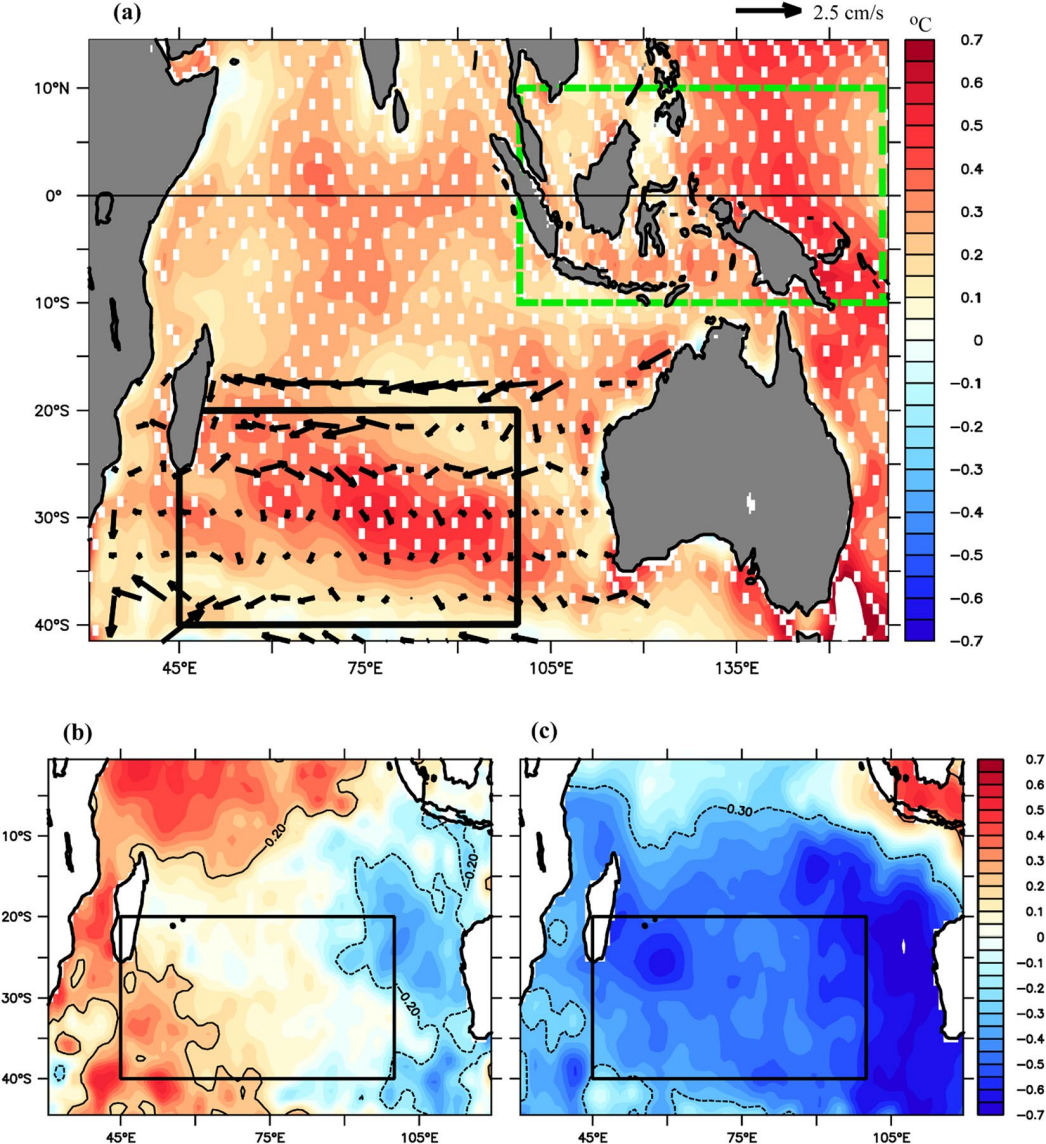
(**a**) SST difference between the GWH (1998–2016) and preGWH (1981–1997) overlaid with the current anomaly (GWH-PreGWH) of upper 100 m. White dots represent the SST anomaly above 90% significance level (**b**), Correlation between MH intensity and SST during the preGWH and (**c**), Correlation between MH intensity and SST during the GWH. The dashed/solid contour indicates 90% significance level. The black rectangle in (**a**–**c**) represent the MH region, and the dashed green rectangle in (**a**) represents the western Pacific region.

To understand the path of heat from the western Pacific (WP) to the Indian Ocean (IO), we examined the spatial SST difference (SST during the GWH - preGWH) overlaid with ocean current anomaly (GWH – preGWH) in the upper 100 m (Fig. 3a). Figure 3a clearly shows that westward flowing south equatorial current (SEC) advecting more heat into the western part of the SIO during the GWH. The latitudinal range of the SEC in the SIO was explored using annual mean surface salinity (ORAS4) overlaid with the annual mean ocean surface currents (Fig. S1). The current anomalies (GWH-preGWH) indicate that during the GWH, westward flowing SEC (Fig. 3a) enhanced at 17°S, where a major volume is supplied by the ITF^22^. Consistent with our result, earlier studies^23,24^ also showed intensification of SEC during the GWH. At the east coast of Madagascar near 17°S, SEC split into northward (Northeast Madagascar Currents-NEMC) and southward branches (Southeast Madagascar Currents-SEMC). The SEMC transports water to the southern tip of Madagascar, and a part of it also retroflects and supplies water to the northeastward flow, east of Madagascar (For a detailed description, please refer Figs. 3 and 4 of Schott *et al*.^18^). The positive SST anomaly in the MH region along with enhanced westward flowing SEC at 17°S indicate that the warming of the region was contributed by the westward-moving currents (SEC) during the GWH (Fig. 3a). The stronger westward current in the SEC path along with larger heat advection^24^ leads to SST increase in the region during the GWH period.

The stronger zonal currents (SEC) are advecting warm water from the WP to the SIO through the ITF^24–27^ which can transport more heat from the Pacific Ocean into the Indian Ocean during the GWH period^28,29^. The westward flowing SEC further advects this warm water to the MH region. Intensification of the ITF during the GWH period helps to advect more warm water into the SIO^29^ with maximum transport through Makassar Strait. Figure S4a,b show the SST anomalies in the western Pacific and the MH region, respectively. Warming of the WP (Fig. S4a) and the MH region (Fig. S4b) is seen during the GWH period. However, the intensification of warming is more in the MH region compared to the WP. Consistent with our result, recent study^30^ also showed the remarkable warming in the upper 700 m of the SIO during the GWH period^11,17^. This was mainly due to the strengthening of the ITF^18,20^, which leads to increased transfer of heat advection from the Pacific to SIO^21^. Spatial maps of temperature advection trend in the mixed layer clearly show an enhanced warming in the MH region during the GWH (Fig. S5b) compared to preGWH (Fig. S5a). This warming leads to acceleration of sea-level rise in the SIO, which is 37% quicker than the global mean-sea-level during the hiatus period^24^. Their study also showed that the ITF heat advection, contribute significantly to the SIO heat budget during the GWH.

**Figure 4:**
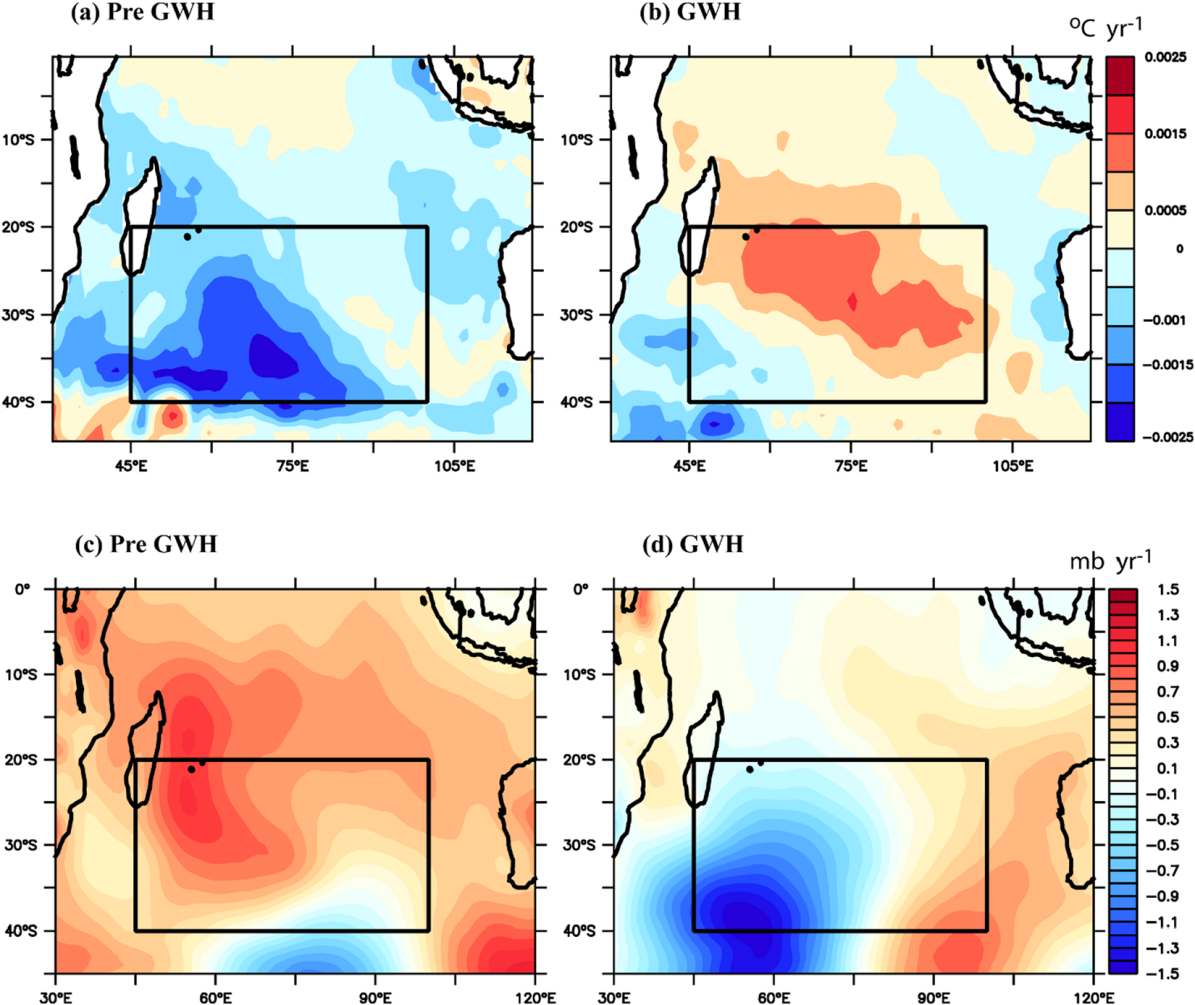
Spatial pattern of trend of (**a**) SST during preGWH, (**b**) SST during GWH, (**c**) MSLP during preGWH, (**d**) MSLP during GWH.

Near 36°S, the Agulhas Current retroflects back towards the Indian Ocean^31^ as the Agulhas retroflection current with some leakage of heat into Atlantic, which may further increase the Atlantic Ocean heat content^32^. This results in the further accumulation of heat in the MH region and reduces the Mean Sea Level Pressure (MSLP) over the region. To understand how these warming affect the intensity of the MH, we have carried out the spatial correlation analysis between SST and MH index during the preGWH (Fig. 3b) and GWH (Fig. 3c) periods. Analysis showed significant negative correlation in the MH region (Fig. 3c) during the GWH whereas no significant correlation observed during the preGWH period. The above result was further explored by analyzing the spatial trends in SST and MSLP during both the periods (Fig. 4). During preGWH, SST showed significant negative trend (Fig. 4a) along with increasing trend in MSLP (Fig. 4c). However, during the GWH, SST showed significant increasing trend (Fig. 4b) accompanied with decreasing trend in MSLP (Fig. 4d). The above analysis confirms that during the GWH, increase in SST reduces the mean sea level pressure that subsequently results weakening of the MH.

**Figure 5:**
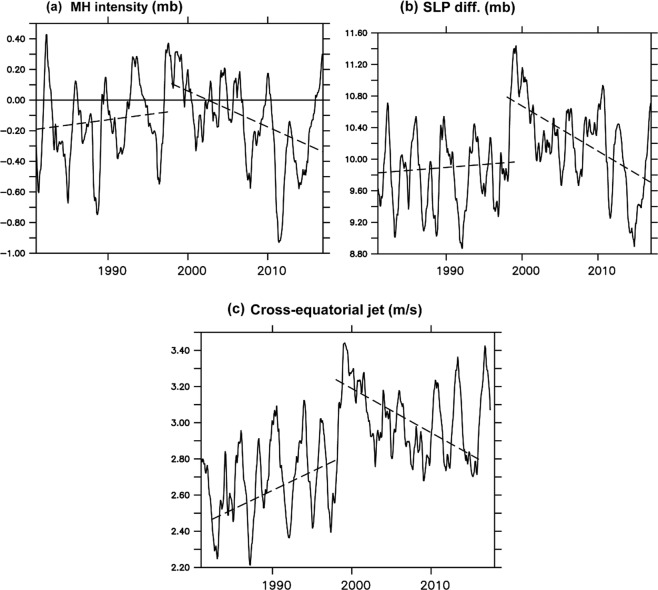
(**a**) Time series of monthly mean SLP, (**b**) SLP difference between the MH [45°E–100°E and 20°S–40°S] and the Indian landmass [45°E–100°E and 10°N–35°N], (**c**) Cross-equatorial winds [37.5°E–60°E and 5°S–5°N]. All trends are significant above a 95% confidence level.

The MH intensity was computed from the mean sea level pressure (MSLP) data using Eq. 4 (Fig. 5a). It showed an increasing trend before 1998 and a decreasing trend after 1998, confirming the weakening of the MH intensity during the GWH period (Fig. 5a). This affects the pressure gradient between the MH (southern hemisphere) and Indian landmass (northern hemisphere) which is a key driver in strengthening the Southern Asian summer monsoon system^33^. Consistent with the MH intensity, MSLP difference also showed a negative trend after 1998 (Fig. 5b), suggesting a significant reduction in the pressure gradient between the southern and northern hemisphere. This further leads to the weakening of the cross-equatorial winds during the GWH period (Fig. 5c). Weakening of this cross-equatorial winds may affect the intensity of the monsoon rainfall over India^34^. All India rainfall showed a decreasing trend during the GWH (Fig. not shown), but not statistically significant above the 95% confidence level. Though earlier studies related the MH intensity and its link with the onset of monsoon over the India^5^, we cannot expect a direct link between the MH intensity and the Indian summer monsoon rainfall. The lack of a significant trend in rainfall was mainly due to Indian monsoon is a complex nonlinear phenomenon involving atmosphere, ocean, and land-based processes^34^. Its intensity is determined or modulated by many factors (for eg., surface heat low, the monsoon trough, the cross-equatorial flow, The Tibetan anticyclone, the Tropical Easterly Jet-Stream), in addition to the MH intensity^7^. Hence it is difficult to correlate one to one relationship between Indian monsoon rainfall and MH intensity. Our study suggests that warming of SST in the MH region leads to a reduction of SLP, and therefore, the SLP gradient between the MH and Indian landmass reduces. The north-south SLP gradient weakens the cross-equatorial winds over the western Indian Ocean.

## Summary

We observed a warming trend in SST in the MH region during the GWH period (1998–2016). This increase in SST decreases the sea level pressure thereby decreasing the pressure gradient between the MH and the Indian Subcontinent. Warming in the WP and intensification of the trade winds during the GWH period leads to the advection of heat to the SIO through the ITF^29^. The strong south equatorial current (zonal current) advects the heat from the WP to the SIO and recirculates this water in the MH region leading to the accumulation of more heat (Indo-Pacific warm water recirculating in the MH region due to the Agulhas retroflection) in the upper ocean, which results in increased sea level and heat content. The resultant warming causes a reduction in mean sea level pressure and reduce the intensity of the MH. This reduces the pressure gradient between the MH and the Indian landmass, which in turn suppresses the intensity of low-level cross-equatorial winds over the western Indian Ocean. For the time, our study reports the influence of the GWH associated changes in the MH intensity and its impact on the cross-equatorial winds in the western Indian Ocean. The weakening of the cross-equatorial winds may affect the strength of the upwelling along the coast of Somalia and Oman and thus impact the Arabian Sea ecosystem.

## Methods

### Mixed layer heat budget analysis

Sea surface temperature (SST) variability in the MH region is studied using the mixed-layer heat budget^14^ for the period 1982–2016. The mixed layer heat in the ocean is mainly influenced by air-sea heat fluxes, horizontal advection, diffusion, and entrainment at the base of the mixed layer. The temperature tendency, i.e., the change occurring in the temperature per month is calculated using the equation,

1 $$\frac{\partial {T}_{m}}{\partial t}=\frac{{Q}_{net}-q(\,-\,{h}_{m})}{{\rho }_{0}{c}_{p}{h}_{m}}-{u}_{m}\cdot {\nabla }{{{\rm T}}}_{m}+\kappa {{\nabla }}^{2}{T}_{m}-\frac{{w}_{e}{\Delta }{T}}{{h}_{m}}$$

In Eq. (1), $\frac{{Q}_{net}-q(-{h}_{m})}{{\rho }_{0}{c}_{p}{h}_{m}}$ represents the air-sea heat flux term, $-{u}_{m}\cdot {\nabla }\,{{{\rm T}}}_{m}$ represents the horizontal advection term (zonal and meridional), $\kappa {{\nabla }}^{2}\,{T}_{m}$ represents the diffusion term, and $-\frac{{w}_{e}{\Delta }{T}}{{h}_{m}}$ represents the entrainment term. The mixed layer depth was computed based on the 0.03 kg m^−3^ density criteria. ^T^m is the mixed layer temperature, ρ_0_ is the reference seawater density which is taken as 1027 kgm^−3^, k is the eddy diffusivity (500 m^2^s^−1^), *C*_*p*_ is the specific heat capacity of water at constant pressure (4000 Jkg^−1^ K^−1^), *h*_*m*_ is the mixed layer depth, *w*_*e*_ is the entrainment velocity, and *ΔT* is the temperature difference between the mixed layer bottom and the layer just below the mixed layer. *q(−h*_*m*_) denotes the downward radiative heat flux at the bottom of the mixed layer. It is based on the assumption of exponential decay with depth^35^. The term is calculated using the equation,

2 $$q(\,-\,{h}_{m})=q(0)[{R}{{e}}^{(-{h}_{m}/{\gamma }_{1})}+(1-R){e}^{(-{h}_{m}/{\gamma }_{2})}]$$

here, q(0) is the short wave radiative heat flux at sea surface and *R*, *γ1*, *γ2* are the constants 0.67, 1 and 17 respectively^14^.

The oceanic advection term ${u}_{m}\cdot {\nabla }\,{{{\rm T}}}_{m}$ (zonal and meridional) was computed using the ORAS4 currents where ${\nabla }\,{{{\rm T}}}_{m}$ is the temperature gradient. The entrainment velocity *w*_e_, is calculated from the equation of continuity for an incompressible flow which is,

3 $$\frac{\partial w}{\partial z}=-\,(\frac{\partial u}{\partial x}+\frac{\partial v}{\partial y})$$

where *u* and *v* are the zonal and meridional currents, respectively.

### MH intensity

In order to compute the MH intensity, the MSLP averaged over the MH region is standardized using the equation,

4 $${\rm{MH}}\,{\rm{intensity}}=\frac{{\rm{MHslp}}-\overline{{\rm{MHslp}}}}{{\rm{Standard}}\,{\rm{Deviation}}}$$

### Total heat content in the Ocean

Total heat content (THC) in the layer from the surface to 200 m depth is calculated for the MH region (45°E–100°E and 20°S–40°S) using the equation

5 $${\rm{THC}}={{\rm{\rho }}}_{0}\,{{{\rm{C}}}_{{\rm{p}}}}^{{\int }_{0}^{h}T(z)dz}$$

where ρ_o_ is the reference density of seawater (1027 kg m^−3^), *h* is the deepest layer of integration (0 to 200 m), and *T* is the potential temperature.

### Cross equatorial winds

The cross-equatorial wind is defined as the meridional wind (V) at 850 mb (or hPa) over the region 5°S–5°N and 37.5°E–60°E^36^.

### Details of data used and sources

Monthly mean SST data is obtained from OI SST for the period 1982–2016 with a spatial resolution of 1 × 1 degree^37^. The monthly means of atmospheric parameters such as Mean Sea Level Pressure (MSLP), long wave radiation, short wave radiation, latent heat flux, sensible heat flux with spatial resolution 1 × 1 degree are obtained from was taken from ERA interim^38^. All the atmospheric parameters were available at https://apps.ecmwf.int/datasets/data. The monthly mean ocean parameters such as temperature, salinity, sea level anomaly, and ocean currents with spatial resolution 1 × 1 degree are taken from ECMWF Ocean Reanalysis System 4 (ORAS4)^39^. The oceanic parameters were taken from http://www.ecmwf.int/products/forecasts/d/charts/oras4/reanalysis/.

Time series of all the anomalies such as SST, heat content, SLA, and mixed-layer budget terms are calculated by removing the climatological monthly means for the period 1982–2016. OI SST data were obtained from https://www.esrl.noaa.gov/psd/data/gridded/data.noaa.oisst.v2.html.

### Comparison of ORAS4 product with other data

In this study, we used ORAS4 products for the mixed layer heat budget analysis. To gain confidence in the data, we have compared the output of ORAS4 reanalysis products with other *in-situ* data. The oceanic parameters such as SST, temperature, and salinity profiles are compared with the gridded Argo data (http://apdrc.soest.hawaii.edu/las/v6) and surface currents with Ocean Surface Current Analyses Real-time (OSCAR; https://podaac.jpl.nasa.gov/dataset/OSCAR_L4_OC_third-deg). Supplementary Figure S2a shows the comparison of ORAS4 SST with OISST and Argo, respectively.

Similarly, vertical structure of climatological temperature (Supplementary Fig. S2b,c), salinity (Supplementary Fig. S2d,e), and time series of zonal (Supplementary Fig. S3a) and meridional (Supplementary Fig. S3b) components of the currents also showed good comparison. The above analysis provides us an assurance that outputs of the ORAS4 products are reliable in the MH region to study the mixed layer heat budget processes. Therefore, the ORAS4 products are preferred for the present study.

### Statistical analysis

All the time series are smoothed with a 12-month running mean, and the trends are computed using least-squares linear regression. Significance of all the trends were tested with two-tailed t-test at 95% confidence interval. All the significant trends are in black font whereas non-significant trends are in bold Italic font in Table 1.

**Table 1:** Trends and errors for the entire variables used for the present study.

Variable	Pre GWH (1981–1997)	GWH (1998–2016)
SST (°C yr^−1^)	−0.006 ± 0.0001	0.02 ± 0.0012
Heat content (*10^8^ Jm^−2^ yr^−1^)	−6.46 ± 0.5	13.27 ± 0.04
SLA (*10^−2^ m yr^−1^)	0.05 ± 0.005	0.470 ± 0.0001
MH Index (yr^−1^)	0.011 ± 0.006	−0.028 ± 0.006
Press. diff (S-N) (mb yr^−1^)	***0.0096 ± 0.0006***	−0.051 ± 0.0006
Cross eq. jet (m s^−1^ yr^−1^)	0.01 ± 0.0001	−0.01 ± 0.0001
Air-sea flux term (°C yr^−1^)	−0.015 ± 0.0012	***0.0012 ± 0.0012***
Advection term (°C yr^−1^)	***−0.0004 ± 0.0002***	0.0066 ± 0.0002

The value, which is not significant above 95% CL, is given in bold Italic font.

## Supplementary information

Supplementary information.

## Data Availability

All data used in this research are freely available and may be downloaded from the links given in the methods section. The codes used for all the analyses are freely available on request to the corresponding author.
