# Author Correction: Global warming hiatus contributed weakening of the Mascarene High in the Southern Indian Ocean

**DOI:** 10.1038/s41598-020-62006-x

**Published:** 2020-03-24

**Authors:** P. J. Vidya, M. Ravichandran, M. P. Subeesh, Sourav Chatterjee, M. Nuncio

**Affiliations:** ESSO- National Centre for Polar and Ocean Research (NCPOR), Headland Sada, Goa 403 804 India

Correction to: *Scientific Reports* 10.1038/s41598-020-59964-7, published online 24 February 2020

This Article contains a typographical error in the Supplementary Information file where the title,

“Weakening of the Mascarene High and its implication to the cross-equatorial winds in the western Indian Ocean”

should read:

“Global warming hiatus contributed weakening of the Mascarene High in the Southern Indian Ocean”

Additionally, in the Acknowledgements section,

“The authors thank Ms Jyoti Jadav, IITM Pune for helpful discussions.”

should read:

“The authors thank Ms Jyoti Jadhav, IITM Pune for helpful discussions.”

