# Supplementary material for: Global warming hiatus contributed weakening of the Mascarene High in the Southern Indian Ocean
Source: Sci Rep. 2020 Feb 24;10:3255. doi: 10.1038/s41598-020-59964-7 (PMC7039868; doi:10.1038/s41598-020-59964-7)
Supplement: Supplementary file 1 — Supplementary information. [file 41598_2020_59964_MOESM1_ESM.docx]

**Supplementary material**

**Weakening of the Mascarene High and its implication to the cross-equatorial winds in the western Indian Ocean**

P. J. Vidya, M. Ravichandran, M. P. Subeesh, Sourav Chatterjee, Nuncio Murukesh

**Figure S1:** Annual mean salinity (ORAS4) overlaid with annual mean surface currents (ORAS4). The black rectangle indicates the domain of the south equatorial current (SEC).

**Figure S2: (**a) Comparison of SST in the MH region (black line: ORAS4, red line: OISST, and green line –Argo SST). Figure b & c indicate the climatological temperature from ORAS4 and Argo respectively. Figure d & e indicate the climatological salinity from ORAS4 and Argo respectively.

**Figure S3:** Times series of **(**a) zonal current from ORAS4 (black) and OSCAR (red line), (b) meridional current averaged over the MH region obtained from ORAS4 (black) and OSCAR (red line).

**Figure S4:** Hovmoller diagram of SST anomaly in the (a) western Pacific (10^o^S - 10^o^N averaged) and, (b) MH (20^o^S - 40^o^S).

**Figure S5.** Spatial maps of temperature advection trend during the (a) preGWH and (b), GWH.

**Supplement Figures**

**
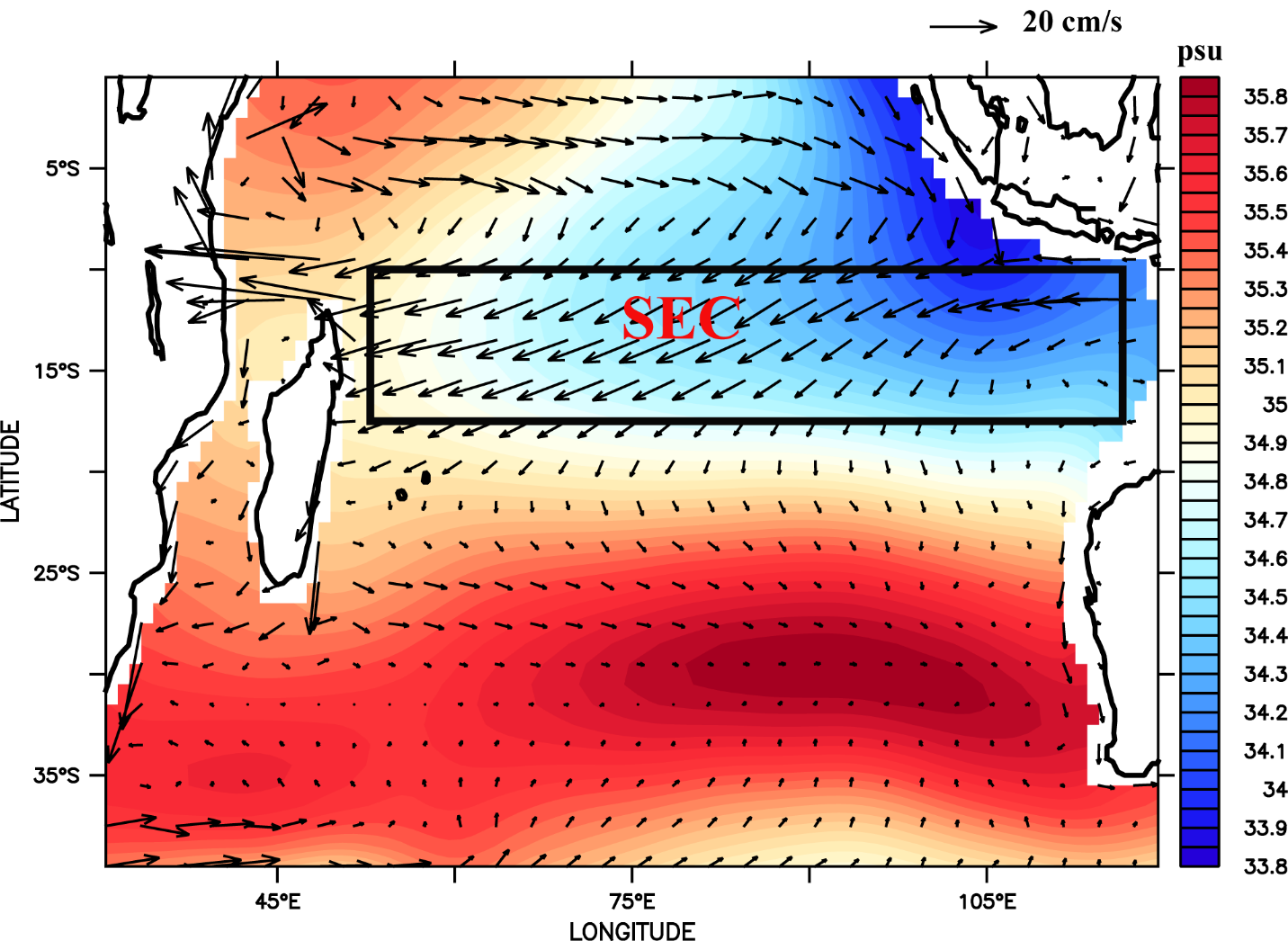
 Figure S1:** Annual mean salinity (ORAS4) overlaid with annual mean surface currents (ORAS4). The black rectangle indicates the domain of the south equatorial current (SEC).

**
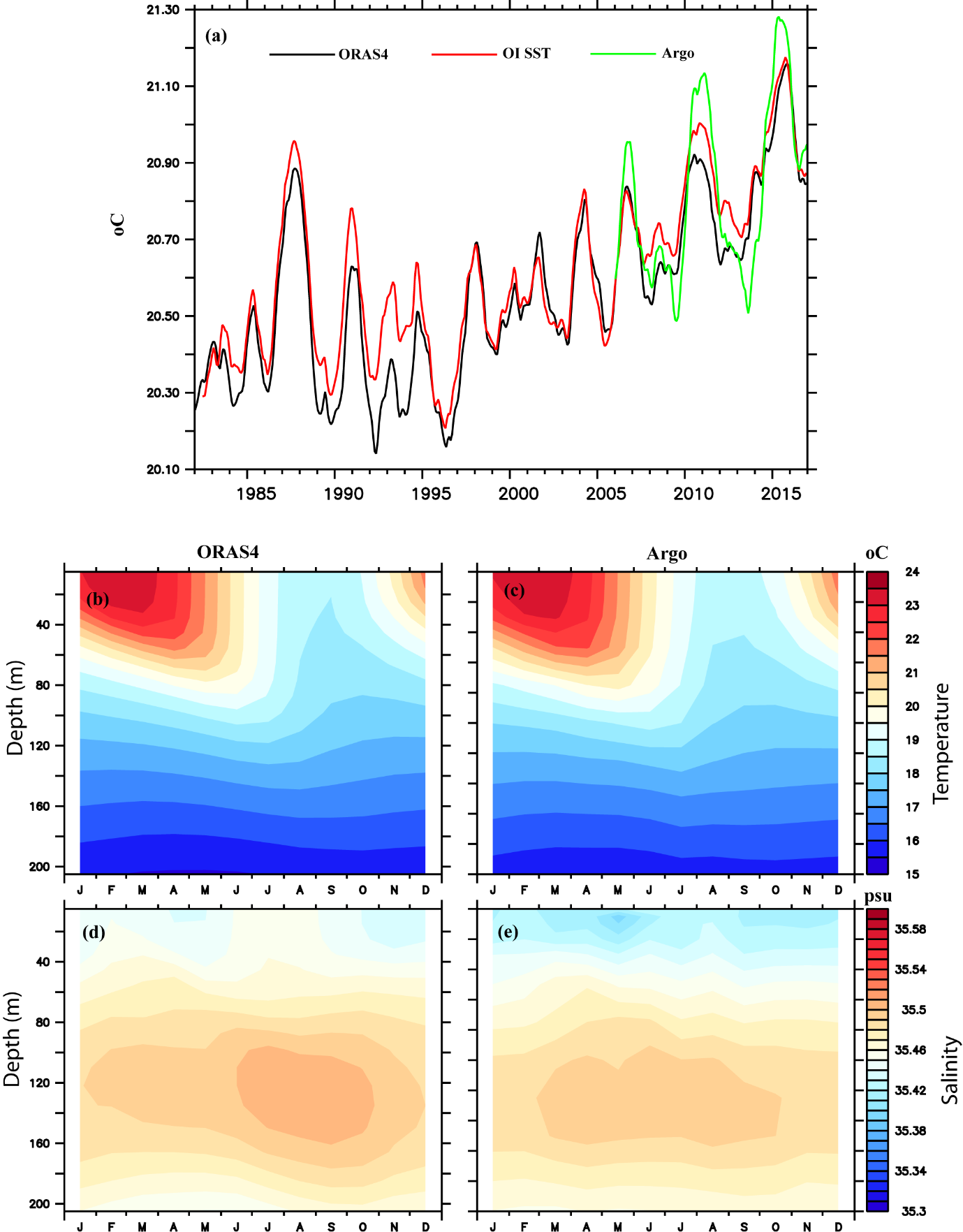
**

**Figure S2: (**a) Comparison of SST in the MH region (black line: ORAS4, red line: OISST, and green line –Argo SST). Figure b & c indicate the climatological temperature from ORAS4 and Argo respectively. Figure d & e indicate the climatological salinity from ORAS4 and Argo respectively.

**
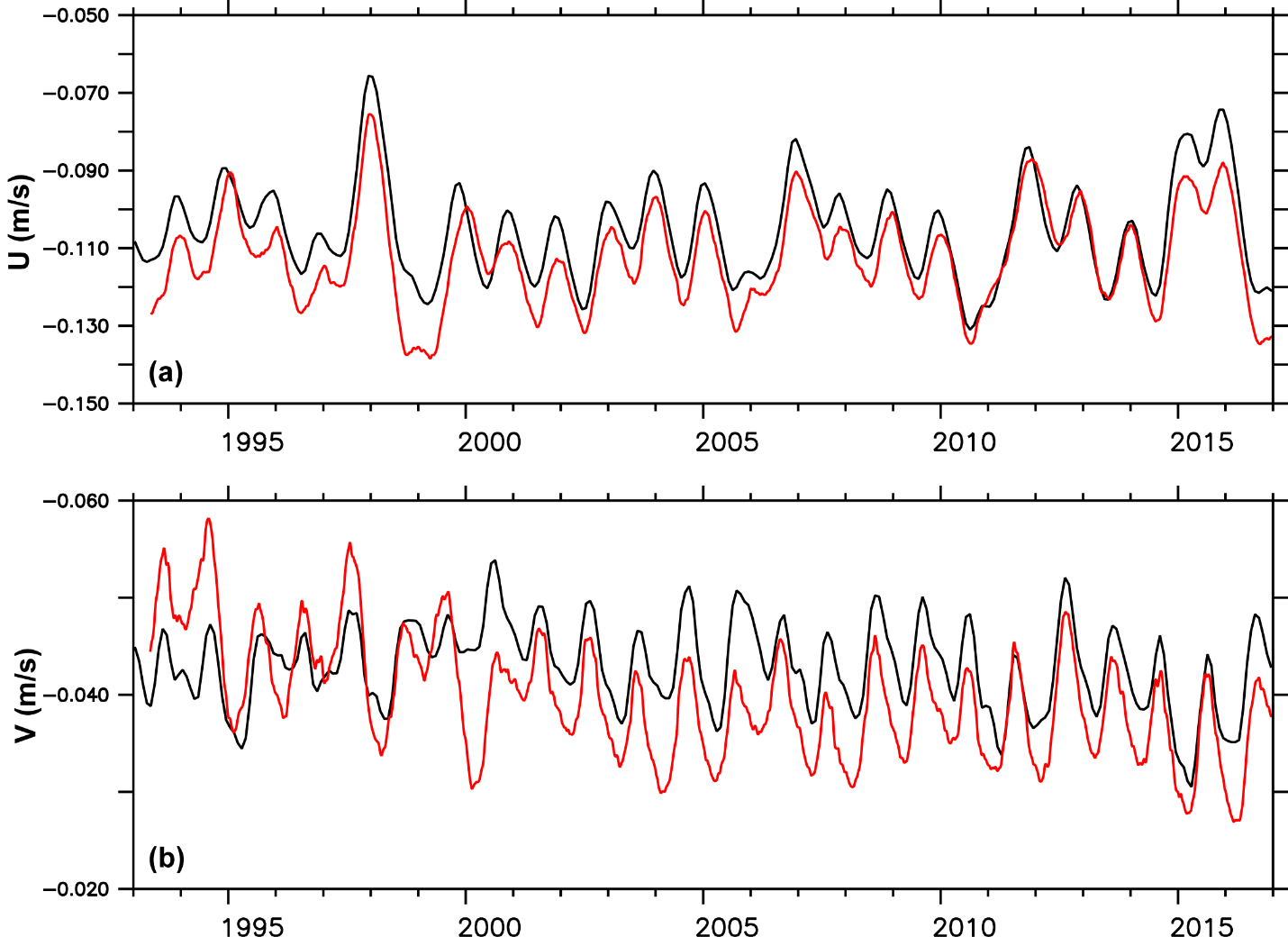
**

**Figure S3:** Times series of **(**a) zonal current from ORAS4 (black) and OSCAR (red line), (b) meridional current averaged over the MH region obtained from ORAS4 (black) and OSCAR (red line).


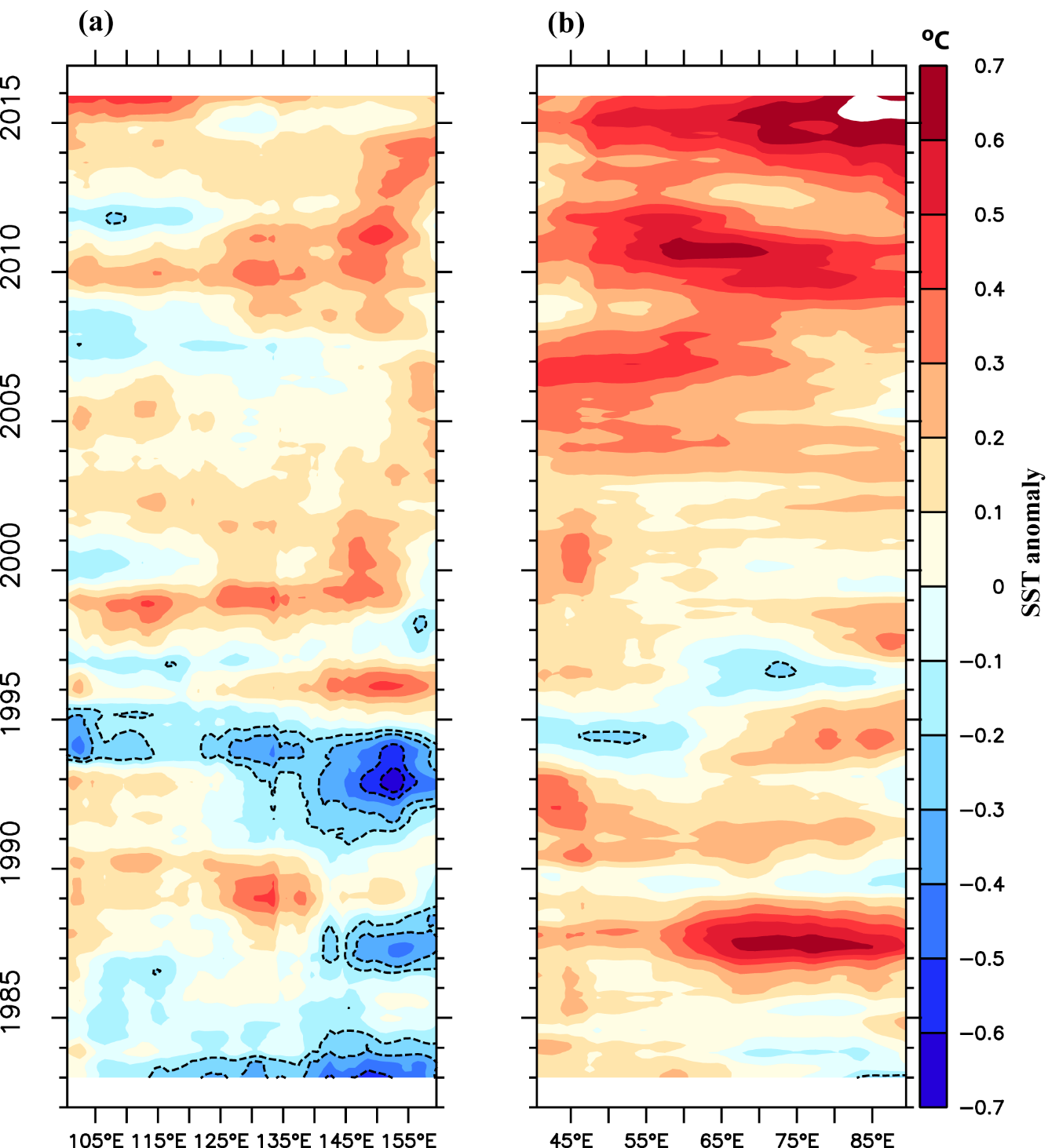


**Figure S4:** Hovmoller diagram of SST anomaly in the (a) western Pacific (10^o^S-10^o^N averaged) and (b), MH (20^o^S-40^o^S).


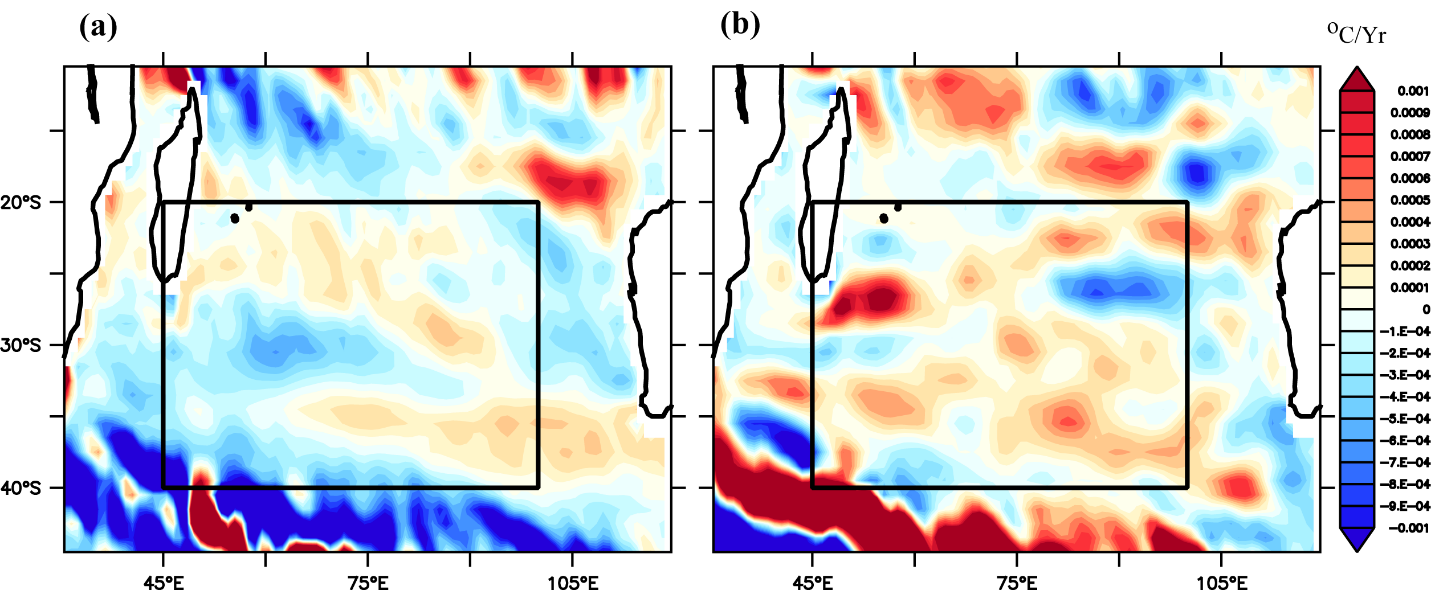


**Figure S5:** Spatial maps of temperature advection trend during the (a) preGWH and (b), GWH.
